# Conditions and factors affecting the accuracy of olfactometric detection

**DOI:** 10.1016/j.heliyon.2024.e41604

**Published:** 2024-12-31

**Authors:** Petra Riedlova, Spiros Tavandzis, Josef Kana, Jaromir Roubec

**Affiliations:** a Centre for Epidemiological Research, Faculty of Medicine, University of Ostrava, Ostrava, Czech Republic; b Department of Epidemiology and Public Health, Faculty of Medicine, University of Ostrava, Ostrava, Czech Republic; c Czech Centre of Signal Animals, Novy Jicin, Czech Republic; d Department of Pulmonary, Vitkovice Hospital, Ostrava, Czech Republic

**Keywords:** Factors, Dogs, Smell, Diagnostics, Olfactometry

## Abstract

**Introduction:**

The use of signal dogs for cancer detection is not yet routinely performed,but dogs and their powerful olfactory system have proven to be a unique and valuable tool for many lineages and are beginning to be incorporated into medical practice. This method has great advantages; the dog can detect a tumour in the human body already in preclinical stages, when the patient has no symptoms yet. The identification of cancer biomarkers to enable early diagnosis is a need for many types of cancer, whose prognosis is strongly dependent on the stage of the disease. However, this method also has its various pitfalls that must be taken into account.

**Aim:**

The aim of the study was to identify and highlight the factors that affect the level of detection accuracy, but also the conditions associated with olfactometric diagnosis.

**Methods:**

The study included 48 dogs and 48 handlers, that were part of the training between 2016 and 2023.All those who started olfactometry training and remained in training for at least one year were included in the study. The dogs ranged in age from 8 months to 12 years and were of different races and sexes. After long-term observation, a qualitative analysis was performed and factors that may play a role in the early detection of the disease were listed.

**Results:**

The results of the search for the different factors have been compiled into two groups, focussing on the actual handling of the patient biological sample from collection, processing, storage until transport, preparation of the sample,and detection. Focus on the actual work and behaviour of the dog and handler.

**Conclusion:**

There are many factors; however, it is worth addressing them because the canine sense of smell is one of the possible uses as a diagnostic method.

## Highlights

• Olfactometry is the science of smell.
• Early diagnosis of tumours is desirable.
• Dogs can diagnose tumours in the preclinical stages.
• There are factors that affect olfactometric detection and therefore need to be addressed.

## 1 Introduction

Olfactometry is a scientific field that deals with the measurement and evaluation of olfactory stimuli, especially the intensity and quality of scents and odours. It is used to objectively assess the olfactory abilities of humans or animals [1]. Canine olfactometry is a method of measuring a dog's ability to detect and distinguish different odours using the sense of smell [2]. It is already commonly used in various professions. Whether it is in forensic science for scent traces, in rescue for searching for people, for finding truffles or, for example, searching for mobile phones in prisons. Signal dogs are also used in healthcare to signal an impending epileptic seizure, hypoglycaemic coma, or to detect various other diseases such as tumours [[3], [4], [5]]. Dog cancer detection has been known since 1989, when a dog first detected melanoma in its handler [6]. Many years have passed since then, and results and opinions on this detection vary.

However, routine use of signal dogs for cancer detection is not routinely carried out; it is still in the research phase. Dogs and their powerful olfactory system have proven to be a unique and valuable tool for many lineages and are beginning to be incorporated into medical practice [4]. This issue has great advantages; the dog can detect a tumour in the human body already in preclinical stages, when the patient has no symptoms yet [4]. This is also evidenced by studies across the world with different success rates (up to 96 %) for detecting different tumours (e.g. prostate, lung, brest tumours), using different biological materials (e.g. breath, sweat, urine, blood) [5,[7], [8], [9], [10], [11]]. The identification of cancer biomarkers to enable early diagnosis is a need for many types of cancer, whose prognosis is strongly dependent on the stage of the disease. However, this method also has its various pitfalls that must be taken into account, and knowledge of them can help both to increase the sensitivity of the method and to clarify possible errors in training, sampling, and sample preparation.

The purpose of the study was to identify and highlight the factors that affect the level of detection accuracy, but also the conditions that are associated with olfactometric diagnosis.

## 2 Methods

### 2.1 Olfactometric detection factors

In the framework of training dogs for the olfactometric detection of selected types of cancer (lung cancer, ovarian cancer) at the Czech Center of Signal Animals (CCSA) in the Czech Republic, after long-term observation between 2016 and 2023 and after the experience of cynologists and researchers in the field, several factors that can play a role in early detection of diseases were written down. These factors were then divided into two groups. Namely, factors that can and do influence the test sample (from collection to olfactometric detection) and factors that influence the training itself (behaviour of the dog, handler).

### 2.2 Criteria for inclusion in the study

The study included 48 dogs and their handlers and were training for a limited time or for the entire 8-year period. All those who started olfactometry training and remained in training for at least one year were included in the study. The dogs ranged in age from 8 months to 12 years and were of different races and sexes. The study was approved by the local Ethics Committee of the Nový Jičín Hospital.

### 2.3 Methodology for training and testing samples by olfactometric detection

**Fig. 1:**
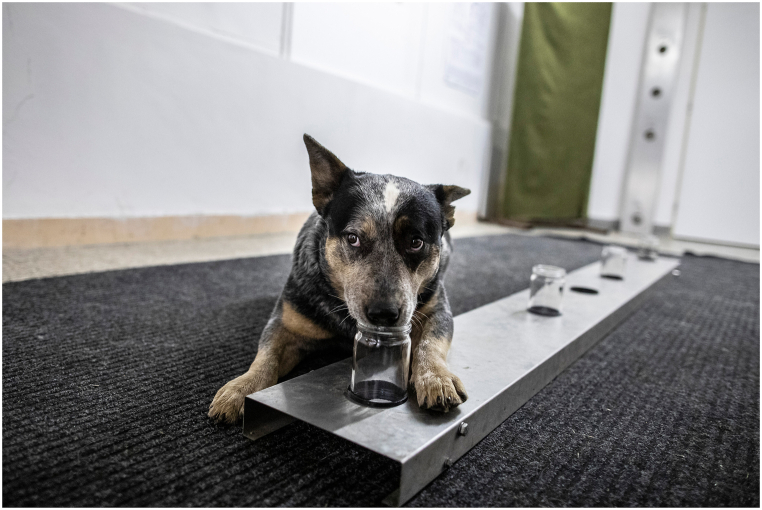
Demonstration of the stainless stell plate and Boolomo the dog at work.

The entire training and testing is a complex system, which is described in detail in a previous article by Riedlova et al. [5]. To simplify and understand the various factors, a brief procedure is outlined below. Blood samples for detection must be processed after collection (serum separation) and stored in a freezer until further use. Samples are specially smelled by dogs in containers (glass) containing odour adsorbers. The odour process is carried out for a minimum of 24 h. The individual adsorbers are then transferred to the scent jars (glass), which are then used to train the dog. Training takes the form of irregular rewards and consists of unblind training and simply blinded tests. Subsequently, double-blind testing also takes place. The trapping jars are placed in the holes of a stainless steel plate, in which a tumour sample (PS) and three samples of the healthy probands (NS) are always placed. In the other case, only NS are placed on the plate. In a double-blind study, three NS and an unknown sample (US) are placed on the plate. The dog tests the samples by sniffing and, when a PS is found, stops at the sample and marks it with a prearranged gesture/movement (e.g. lie, sit, bark) (Fig. 1) [5].

## 3 Results

The results of the search for the different factors that affect the entire olfactometric diagnosis have been compiled into two groups shown in Table 1, Table 2. The first group (Table 1) focusses on the actual handling of the patient/proband's biological sample from collection, processing, storage until transport, transport, storage in CCSA, preparation of the sample for odour work to canine scent testing.

**Table 1:** Factors that affect the sample for olfactometric diagnosis.

Sample pathway	
*Medical equipment → CCSA*	*Description of individual factors*
***Sampling from the proband***	same type of sampling tubes for PS, NS and US
***Sample information***	sex
	age
	other, associated diseases
	blood group
	type of tumour
	tumour grade
	medication used
***Sample preanalytics***	collection time (before surgery, before the start of chemotherapy)
	day of collection
	method of storage in the medical equipment
	transport
	processing in the laboratory
	storage at the CCSA
	associated environmental odours
***Protection against sample contamination (microbiological, odour)***	separate preparation of PS and NS
	working with protective equipment (gloves, tweezers)
	introduction of foreign odours (environment)
	washing of odour and training jars
	temperature
	humidity
	depth of freezing
***Working with the sample in the Centre (researchers)***	storage
	quantity and method of smelling
	introduction of foreign odours (during preparation)
	time of curing
	period of use
	dusting
***Odour containers***	shape of the can
	sample volume
	number of swabs
	material (glass/plastic/stainless)

**Table 2:** Factors that affect the dog and handler in the olfactometric diagnosis.

Training and olfactometric detection	
*Test sample in practice*	*Description of individual factors*
***Dog and handler***	sniffing (introductory sample)
	breed/age
	faults (dog/species)
	mood of the dog
	type of exercise (assisted/self-training)
	position of sample
	number of samples to be trained
	knowledge sharing
	own thinking and reasoning
	rewards (positive motivation, irregular rewards)
	rituals
	concentration
	room temperature and humidity
	load (amount of practice samples)
	exercise intensity (daily, weekly, monthly)
	exercise (rhythm, home/centre, time of day, strange dogs/exercise, before/after feeding).
***Work sample and training***	sex
	samples from odour cans- separate, mixed
	dripped working samples
	age of sample (freshness, 10–15 days)
	introduction of foreign odours (during exercise)
	duration of odouring and use
	number of times the working sample has been opened
	number of times the containers have been opened
	amount and method of smelling
	duration of odouring
	duration of use of a single sample
	probability of a negative sample
	ambient odour

The second group (Table 2) focusses on the actual work and behaviour of the dog and handler during olfactometric detection. Some of the factors are present in both tables due to their influence on both the sample itself and the dog's training.

Once the above requirements have been met and adverse effects have been reduced, i.e., negative factors affecting training have been avoided, the actual research and testing can begin. Each of the above factors can play a large role in the training itself, but also in the interpretation of the results.

## 4 Discussion

Olfactometric detection has been known for more than 30 years [6]. Dogs have a detection sensitivity better than any diagnostic device [5]. However, the dog is a living creature, so there are many factors that can influence detection, and sensitivity can be variable. Another problem is that it is still not known exactly which molecules, or combinations of molecules, dogs respond to Refs. [5,7]. This is perhaps one of the reasons why olfactometric diagnosis is still not a recognised official method. However, if the correct techniques and procedures are followed, it is more than worthwhile to engage in this methodology because early cancer diagnosis, even if it would benefit for only a one patient, is very useful. The advantage of this potential screening is its noninvasiveness and low cost detection [7].

Blood samples for olfactometry are collected in hospitals, blood collection centres, or by outpatient doctors. Here a problem may occur, namely the use of different types of sampling tubes. However, this potential problem has not yet been resolved. The solution is to have a collection site with one type of tube, but this makes it difficult to collect blood in more remote areas where the probands come from.

From almost eight years of observation, it was found that “basic sample information” is unlikely to affect olfactometric detection. From a previous study by Riedl et al. the test group was of different ages, blood types, at different stages of disease, with different types of lung cancer and taking different types of medication. The results show that it is in some ways more attractive for dogs, as evidenced by the amount of sensitivity of the method [5,12]. Since no personal medical history is available from the physicians except to determine sex, age, tumour type, and stage, we believe that these factors do not affect the training itself [5]. However, what undoubtedly plays a role is the time of collection samples. If patients were sampled during chemotherapy, then the dog could be confused by such samples [4,13]. Therefore, patient samples must be used for training before starting treatment. The sex of the probands may also be a confounding factor. This is not a problem in the detection of lung cancer [5,12], but sex could play a significant role in the detection of ovarian cancer. If we put a sample from a healthy male among the samples from females, the dog could mislabel this sample because it is different from the other samples. The final factor from the "sample information" group (Table 1) that is probably the biggest problem is to use a sample from a "potentially healthy person" and pass it off as negative. Although verified tumour samples are considered NS, it is never 100 % that this sample no longer has tumor molecules in it. We present it to the dog as non-tumour, and this can repeatedly confuse the dog.

In order to minimise and exclude negative factors such as the influence of for example smoking, alcohol, coffee, garlic [4], we specialise only in blood [5,12,13]. Dogs can detect tumours from other biological materials with the same accuracy (e.g. breath, sweat, urine), as shown in other studies [5,7,8,11,13,14], however, blood is the most stable and suitable for these purposes under our experience and in our conditions.

When processing the sample itself, care must be taken to process it within 8 h after collection and to store it in a freezer at a minimum of −20 °C after serum separation. Long-term observation has shown that the samples can be used for several years after these conditions are met [5]. The transport of samples to the CCSA is also frozen state. Its further storage must be free of other associated ambient odours. Protection against sample contamination is one of the most important aspects of the whole system. It is important to avoid contamination of positive (tumour) and negative (non-tumour) samples, hence a separate sample preparation room is offered. What cannot be fully adhered to is the preparation of unknown samples which are used for double-blind testing. Therefore, an increased level of caution is required. To avoid contamination of samples, it is necessary to work with protective equipment (gloves, tweezers) must be used in an appropriate laboratory room without the presence of excess foreign odours from the surroundings. Proper and sufficient washing of the odour containers is essential, unless it is possible to use new containers at all times. Both the sample in the container and subsequently in the jar are significantly affected by the temperature and humidity of the surrounding environment. These factors also seem to be some of the most difficult to maintain. CCSA has developed a special system for the method of evaporation, determining the amount of sample used, as well as the number of adsorbers used in the jars. The period of time to smelling the sample and use is currently being tested, but at this time it is confirmed that the sample must be smelted for a minimum of 24 h [5]. It is important to find out how long the sample is able to "feel", how long it can be used, and when it needs to be replaced. Most studies use a dripped (direct) fresh sample for training, i.e. not an adsorber [11,13]. However, in our case, this appears to be suboptimal. It has been observed that when the dog does not smell such an intense odour, it is more pleasant and clearer for him. It has also been tested that when the dog is given a sniff of a fresh odour adsorber taken out of the container, it does not notice it. It is necessary to let it air out for a few hours. Last but not least, it is important to have similar, preferably identical odour cans. We assume that the shape will not play a role, but that the aforementioned sample volume, container size, and number of odour adsorber inserts will. Due to the fact that we have developed this odour canning system ourselves, there is no way to obtain this valuable information from other studies and it is necessary to test this [5]. However, it is clearer than ever that the repeated use of plastic for odour purposes is absolutely inappropriate.

Another group of influencing factors is the training itself. It is essential to let the dog sniff correctly and at the optimal length, both by using the so-called introductory sample and by starting the training with the already known PS and NS, so that the dog is confirmed of what to look for later. The breed of the dog plays almost no role. However, dogs with a short muzzle length, the brachycephalic type, are not suitable; the mesocephalic type muzzle is most suitable [15].

Due to the length of the dog's learning process, it is advisable to choose a young dog, but one that has already mastered the basic commands. Working with him is then easier, and he will sooner understand what is required of him. Choosing a young dog is also because of its useful life and therefore longer use for trained diagnostic dogs. But that is the only reason to be concerned about age. We already know from experience that a nine-year-old dog responds equally well to learning and training. What matters more than the experience and age of the dog is the experience of the handler. Even if the dog is the smartest, if the right guidance is not given, the result will not occur. It is therefore important to focus not only on the faults of the dog but also of the handler, and especially on the interaction between them. Much emphasis is placed on unconscious cues from the handler towards the dog, whether verbal or non-verbal (unconscious movements, gestures) [16,17]. This is a big and common problem in training, but it is completely eliminated in double-blind testing. The performance of the dogs, but also the correct training technique, must be systematically checked by the aforementioned double-blind test [7].

Another influencing factor is the form of training, which takes place individually. It makes a big difference if the handler trains alone with his dog or together with another assistant who prepares the combinations and positions of the inserted samples. He combines the samples differently than a handler training a dog would. The handler tends to repeat the insertion of the positive sample in the same positions at certain intervals, or to cue the dog unconsciously.

Important, if not most important, is the number of samples available for training. The higher the number of samples used, the lower the dog's ability to remember them (ideally, higher tens to hundreds of samples). Samples can be used repeatedly for training, but there must be a sufficiently long interval between repetitions, at least six months [5]. Another factor is the mutual sharing of knowledge around training. Although training is slightly different for each handler and dog, new stimuli are important to improve performance and increase detection sensitivity, as is the use of "own brain" and thinking during individual training sessions.

Positive motivation and regular/irregular rewards play a very important role in training. Irregular reward is one of the most difficult and complex things to do when working with dogs. For the dog, training must be a game that ends with a reward that is appropriate for the dog (e.g., treat, toy) [17]. After intense exercise, which is very mentally demanding for the dog, he must have enough space to rest and regenerate. However, the intensity of training is very individual and it is important, but at the same time very difficult, to estimate how often and for how long it is optimal to train. Some dogs can be training three times a day, others once a day, some maybe only once a week.

Another point in training is rituals. Every dog needs to know in advance that he is now going to "work". At that moment, the dog starts to look ahead and prepares for the training. Rituals can be introduced before, during or after training. The handler must know his dog perfectly to know when he is no longer focused on the work and just wants to do it in some way. Then he must positively end the training at the appropriate time.

Some dogs are also very sensitive to the environment in which they train. There are dogs that need to be alone when working, but there are also dogs that don't mind more people or other dogs. They either train only at home, or at a training centre, or a combination of the two. Every handler should know when it is best for his dog to train [5,7], at what time of day or night, and whether before or after feeding. And the most important thing, which unfortunately belongs to uncontrollable factors, is the mood of the dog. A dog has its days, it may be sick, it may not be willing to work, and if we forced such a dog to train, the results would not come [7,16].

The same way the sample itself can be affected or degraded and rendered unusable for work, so the sample may be degraded or rendered unsuitable for the dog during training. Most of the factors are already intertwined from Table 1, such as sex, fresh working samples, and their intensity, the likelihood that a negative sample is actually negative, or smelling time in containers. The number of times the smelling jar (but also the smelling containers) was opened due to odour drift, the surrounding environment and the possibility of odour intrusion from the environment may also play a role in training. However, odours can also affect the dog during training. This should take place without any distractions, e.g. food in the vicinity, treats elsewhere than with the handler. The introduction of odour (most often a treat) into individual samples could also be a problem. If this happens, the sample should be disposed of immediately and not used for training. Not only odours, but also ambient temperature and humidity are important for training. Dogs, but also their sense of smell, are affected by higher temperature and humidity [18,19]. At higher temperatures, the dog's homeostatic compensatory reaction increases, and it has to breathe more to cool down. This reduces and compromises his olfactory senses, but also reduces his performance and ability to work [4,18].

The same samples in the same jars are repeatedly used for training, but the length of use varies between handlers. There may be a perception that the dog may mark the positive jar, and therefore it is not advisable to use it again for training. Due to the fact that the dog does not have a 100 % success rate in training, it happens that he marks the NS (marks it) as PS. Therefore, in the next round it would be assumed that he will return to it because he has marked it before. However, this does not happen. Thus, his marking the sample does not have an effect. So if the dog marks the samples, then both NS and PS. However, reuse of the same samples is not done in double-blind testing. There, new samples and jars are always used.

The last factor affecting the dog's training is the separate (single) samples, but also the mixed samples. Just as the pooling of samples takes place in laboratory work, a similar pooling could be done for the single PS or NS. This is still in the testing phase, but preliminary results show that it is much more difficult for the dog. However, perhaps if it works, it would speed up training. A study by Hall and Wynne indicates that dogs trained with mixtures tend to perform better than dogs trained on pure scents. However, these results were related to chemicals [20]. Theoretically, this could be similarly applicable for olfactometric detection purposes.

Everything that has been investigated here is a very individual matter, but also very important and valuable information to ensure that the results of dogs are of good quality with ever increasing sensitivity. Adherence while eliminating negative factors can improve the whole work of the dog and handler. We expect that knowledge of these factors will not only influence, but also speed up the entire training and diagnosis.

## 5 Conclusion

The olfactometric diagnosis is not as simple as it may seem at first sight. There are many factors that can influence both the sample itself and the whole training. However, it is worth addressing them because the canine sense of smell is one of the possible uses as a diagnostic method. This is valuable material for further elaboration and expansion of possible factors so that the olfactometric diagnosis is at the highest possible level.

## CRediT authorship contribution statement

**Petra Riedlova:** Writing – original draft, Resources, Project administration, Methodology, Formal analysis. **Spiros Tavandzis:** Writing – original draft, Resources, Methodology, Data curation. **Josef Kana:** Supervision, Investigation, Data curation. **Jaromir Roubec:** Supervision.

## Ethical approval

The study was approved by the Ethics Committee of Nový Jičín Hospital, Czech Republic. As this is not animal testing but collaboration with animals, no application has been made to the Animal Ethics Committee.

## Précis

It is very important to consider olfactometric diagnosis as one of the screening methods. Early detection of tumours is essential for the outcome of treatment.

## Data availability statement

The raw data supporting the conclusions of this article will be made available by the authors, without undue reservation.

## Declaration of competing interest

The authors declare that they have no known competing financial interests or personal relationships that could have appeared to influence the work reported in this paper.
